# Comparison of heterogeneity quantification algorithms for brain SPECT perfusion images

**DOI:** 10.1186/2191-219X-2-40

**Published:** 2012-07-20

**Authors:** Romain Modzelewski, Elise Janvresse, Thierry de la Rue, Pierre Vera

**Affiliations:** 1 Laboratoire d’Informatique, de Traitement de l’Information et des Systemes (EA-LITIS 4108), QUANT. I. F. (Quantification en Imagerie Fonctionnelle, Faculty of Medicine, Rouen University, Saint Etienne du Rouvray, 76801, France; 2 Department of Nuclear Medicine, Henri Becquerel Center and Rouen University Hospital, Rouen, 76000, France; 3 Unite Mixte de Recherche – Centre National de la Recherche Scientifique (UMR-CNRS 6085), Raphael Salem Mathematics Laboratory, Saint Etienne du Rouvray, 76801, France; 4 Henri Becquerel Center, Nuclear Medicine Department, 1 rue d'Amiens, Rouen, 76000, France

**Keywords:** Heterogeneity, Quantification, Functional imaging, Brain, Single photon emission computed tomography, Perfusion

## Abstract

**Background:**

Several algorithms from the literature were compared with the original random walk (RW) algorithm for brain perfusion heterogeneity quantification purposes. Algorithms are compared on a set of 210 brain single photon emission computed tomography (SPECT) simulations and 40 patient exams.

**Methods:**

Five algorithms were tested on numerical phantoms. The numerical anthropomorphic Zubal head phantom was used to generate 42 (6 × 7) different brain SPECT simulations. Seven diffuse cortical heterogeneity levels were simulated with an adjustable Gaussian noise function and six focal perfusion defect levels with temporoparietal (TP) defects. The phantoms were successively projected and smoothed with Gaussian kernel with full width at half maximum (FWHM = 5 mm), and Poisson noise was added to the 64 projections. For each simulation, 5 Poisson noise realizations were performed yielding a total of 210 datasets. The SPECT images were reconstructed using filtered black projection (Hamming filter: α = 0.5).

The five algorithms or measures tested were the following: the coefficient of variation, the entropy and local entropy, fractal dimension (FD) (box counting and Fourier power spectrum methods), the gray-level co-occurrence matrix (GLCM), and the new RW.

The heterogeneity discrimination power was obtained with a linear regression for each algorithm. This regression line is a mean function of the measure of heterogeneity compared to the different diffuse heterogeneity and focal defect levels generated in the phantoms. A greater slope denotes a larger separation between the levels of diffuse heterogeneity.

The five algorithms were computed using 40 99mTc-ethyl-cysteinate-dimer (ECD) SPECT images of patients referred for memory impairment. Scans were blindly ranked by two physicians according to the level of heterogeneity, and a consensus was obtained. The rankings obtained by the algorithms were compared with the physicians' consensus ranking.

**Results:**

The GLCM method (slope = 58.5), the fractal dimension (35.9), and the RW method (31.6) can differentiate the different levels of diffuse heterogeneity. The GLCM contrast parameter method is not influenced by a focal defect contrary to the FD and RW methods. A significant correlation was found between the RW method and the physicians' classification (*r =* 0.86; *F =* 137; *p <* 0.0001).

**Conclusions:**

The GLCM method can quantify the different levels of diffuse heterogeneity in brain-simulated SPECT images without an influence from the focal cortical defects. However, GLCM classification was not correlated with the physicians' classification (*Rho = −*0.099). The RW method was significantly correlated with the physicians' heterogeneity perception but is influenced by the existence of a focal defect.

## Background

Measuring the heterogeneity of medical images has been described for many medical conditions, including in bone marrow to quantify osteoporosis [1,2], for tumor characterization [3-5], myocardial metabolism with positron emission tomography (PET) [6-10], in subjects with Alzheimer’s disease (AD) with PET [11], perfusion imaging in brain single photon emission computed tomography (SPECT) [12-14], and perfusion in brain SPECT images in patients suffering from AD [15] or from drug-induced therapy [16].

Heterogeneity is a visual perception that varies by person. In clinical practice, physicians subjectively analyze the heterogeneity of medical images because of the lack of an objective and reliable method to quantify this parameter. In functional imaging, such as nuclear medicine, the analysis of image heterogeneity is complex because of a low signal-to-noise ratio and low spatial resolution. Artifacts from the reconstruction process are another type of noise superimposed on images dedicated to the exam's interpretation. Because of this subjective aspect, quantification and analysis of the heterogeneity of medical images are complex and less reproducible. An ideal mathematical method would be reproducible, objective and would provide results that are correlated with the physician's results. Some methods have been described in the literature to solve the problem of a varying analysis of heterogeneity in medical images. These methods are mostly based on a fractal analysis [15,17], coefficient of variation calculation [11], and texture analysis [18-23]. Recently, descriptors based on the random walk (RW) theory have emerged [24-28]. Each of these methods is derived from different mathematical theories and yields a different view/representation/perception of an image.

Functional imaging of the brain with 99mTc-hexamethyl-propylene-amine-oxime (HMPAO) or 99mTc-ethyl-cysteinate-dimer (ECD) SPECT assesses cerebral blood flow (CBF). A CBF measurement with SPECT is valuable for most cerebral diseases, particularly in patients with AD [29,30] or epilepsy [31,32]. In these pathologies, SPECT abnormalities are focal, and different methods have been developed to quantify the intensity of focal CBF abnormalities.

Diffuse brain SPECT abnormalities have been described in most systemic diseases, such as systemic lupus erythematosus [33,34]; hypothyroidism [35]; after chemotherapy [16,36], carbon monoxide poisoning [37] or manganese toxicity [38]; and in cocaine [39] or alcohol abuse [17]. Because objective quantification is lacking, brain SPECT images were analyzed visually in these previous studies.

The present paper compares our method, which was based on the RW method, with the five methods commonly used in the literature to evaluate the heterogeneity of SPECT images. The comparison was first conducted with simulated brain perfusion SPECT images to assess how the images are influenced by focal cortical defects (such as in AD) and the global diffuse heterogeneity. The goal was to find the best algorithm to discriminate the more diffuse perfusion heterogeneity levels without an influence from focal defects. The second part of the manuscript will compare the classification of these algorithms using real brain HMPAO SPECT perfusion images. The population was composed of 15 normal subjects and 25 patients referred for memory impairment. The goal of this portion of the study was to find the best algorithm that correlated with the physicians' consensus.

## Methods

To evaluate the RW algorithm, a comparison of five traditional algorithms was performed on phantom and real brain perfusion images. Algorithms were computed only on the cortical rim, obtained with a masking process (described in § 2.2.2) A brief description of each method is presented below:

1 The coefficient of variation (CV) is defined as the ratio of the standard deviation and the mean of the intensity levels. In our case, the CV was measured in the whole brain slice (CV3D)

2. The entropy, *H*, is expressed as:

(1) $$H=\sum_{i=0}^{G-1}p(i)\log_{2}[p(i)]$$

where *p(i)* is the density probability of the gray-level occurrence *i*, and *G* is the number of gray levels in the image. Entropy was first calculated as a global 3D parameter (entropy 3D). Entropy was also computed as a 2D parameter as follows: each pixel was replaced by the measure of the entropy of its neighborhood (3 × 3 and 5 × 5); the sum of all local entropies in all brain slices represents the final result (entropy 3 × 3 and entropy 5 × 5, respectively).

3. The fractal dimension (FD) followed two algorithms: (1) the box counting (BC) method [40] and (2) the Fourier power spectrum (FPS) method [41].

The 2D FD BC method analyzes the image by the complexity changes in its different gray-level and uses a set of threshold values *T* (0.3 *< T <* 0.7) to achieve a set of binary versions of the original image. The fractal dimension is estimated by the slope of the regression line between the size of the boxes (1, 2, 4, 8, and 16 pixels) and the number of the boxes needed to encompass the binary object. In this case, the minimum, maximum, median, mean, and standard deviation were studied along slices.

The Fourier power spectrum method or FD FPS calculated in 2D (mean of the FD along the slices) is based on the power spectrum dependence of the fractional Brownian motion. In the 2D power spectrum method, each line height profile that forms the image is Fourier transformed; the power spectrum is evaluated and all of these power spectra are averaged. The fractal dimension is evaluated from the slope, *β*, of a least-squares regression line fit to the data points in a log-log plot of the power spectrum, as FD = 7/2 − *β*/2. The FPS was also calculated in 3D (FD FPS 3D). Reader is referred to [40] for a detailed description of the FD FPS in 2D and its generalization in higher dimensions. Algorithms are derived from the original implementation in [42].

4. In the gray-level co-occurrence matrices method (GLCM) [43,44], each matrix represents the joint probability distributions between the gray-level values of pairs of pixels at a predetermined distance (1 pixel) and orientation (0°, 45°, 90°, and 135°). The GLCMs were averaged to obtain one GLCM for each 2D slice. Four statistical measures or parameters of the GLCM (i.e., contrast, homogeneity, energy, and correlation) were computed on 255 gray levels. A fifth parameter averaging three spatial distances (1, 2, and 3 pixels) was also created.

5. In the random walk method or RW [27,28], a virtual walking particle performs a random walk on a 2-dimensional image lattice with a four-neighbor system. The purpose of the RW is to visit a large number of new pixels in homogenous regions and to be confined (i.e., to pass over the same pixels previously visited) within heterogeneous regions. Each step of the RW is decomposed into the following two procedures:

(a) Moving procedure

When (i, j) is the current position of the particle, the decision to move or not with a probability (P_move_) is described by the following expression:

(2) $$P_{\text{move}}(i,j)={\left(\frac{I(i,j)}{\max(I(x,y))}\right)}^{S}$$

where *I*(i,j) is the intensity of the pixel, (i,j), the denominator is the maximum intensity taken over all pixels *(x,y)* from the selected slices, and *S* is a slowing parameter *(S =* 3*)*. The purpose of this procedure is to differentiate the behavior of the particle according to the local intensity. A number, *q*, is randomly chosen between [0, 1]. If *q < P*_move_, the movement is accepted, and the algorithm continues to the jump procedure. If *q ≥P*_move_ and the movement is not accepted at time *t*, the particle stays at its current position and a new attempt is made at time *t +* 1.

(b) Jump procedure

This procedure is performed only if the movement has been previously accepted. If so, the algorithm decides which of its four neighbors the particle will jump to according to the following transition probabilities. The probability of jumping from a pixel (i,j) to one of its neighbors (k,l) is given by the following expression:

(3) $$P_{\text{jump}}\left\{\left(i,j),(k,l\right)\right\}=\frac{\exp(I(k,l)/T)}{\sum_{\left(m,n\right)}^{}\exp(I(m,n)/T)}$$

where the denominator is the sum taken over the four neighbors (m,n) of pixel (i,j), and *T* is the temperature parameter. The higher *T* results in an easier jump from high intensity (hot) regions to low intensity (cold) regions. We experimentally chose *T =* 6.

After 1,000 steps, the algorithm outputs the visiting rate denoted *r*, which is the number of visited pixels divided by 1,000. To provide a good estimation of the expected value of *r*, a large number (1,000) of independent random walks on the 2D-image are computed. This procedure is repeated on a selected number of slices in the cortex. At the end, the algorithm outputs *R*, the average of *r*-values over all the selected slices. *R* is the average number of visited pixels as a percentage (*R* value in percent). The reader is referred to [27] for a full description and validation of the RW algorithm parameter.

### Phantom study

To study the robustness of the algorithms and their ability to discriminate between diffuse heterogeneity and a focal defect, a simulation experiment was performed using MATLAB, v 7.0 (Mathworks, Natick, MA, USA) on a standard personal computer platform.

#### Phantom design

**Figure 1:**
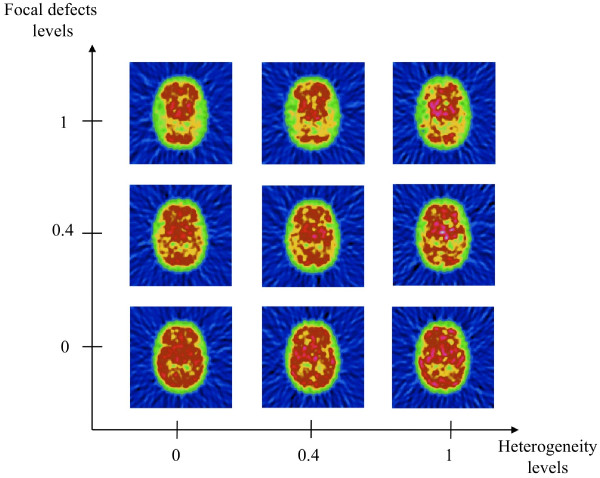
**Examples of nine samples from the 210 non-masked numerical Zubal phantoms are represented.** The diffuse heterogeneity level and the focal defect level were normalized. Zero is a normal level, and one is the most abnormal level.

Simulations of normal and pathological phantom are presented in Figure 1.

##### Normal phantom

The modified Zubal head phantom [45] was used to simulate normal HMPAO brain perfusion SPECT images. First, the 63 anatomical regions of the magnetic resonance imaging (MRI)-Zubal head phantom were transformed into 19 functional regions. The cortical, extra-cortical, and extra-cerebral blood flow values were obtained from a normal database of 12 controls (8 women and 4 men; mean age ± standard deviation (SD), 74. ± 3.9 years) followed in the prospective Eugeria study [46]. Morphologic imaging (magnetic resonance imaging and computed tomography) was normal in the 12 control subjects. The CBF values were obtained from Catafau et al. [47], and the methodology has been described previously [48]. The cerebellum CBF value was arbitrarily defined as 100. Effects of sex, interhemispheric asymmetry, and age were not taken into account with this model.

##### Pathological phantom

Diffuse brain heterogeneity and focal perfusion defects were successively simulated. Diffuse heterogeneity of brain perfusion was simulated by adding Gaussian noise only in the cortex region. This Gaussian noise has an adjustable variance and a zero mean. Six diffuse heterogeneity levels were constructed (σ = 0 for normal subjects, and successively σ = 100, 2,000, 4,000, 6,000, 8,000, 10,000 for the six levels of heterogeneity). The focal defect simulation was used to mimic Alzheimer’s disease, which is characterized by temporoparietal (TP) defects. Five different levels of TP perfusion were used (93 for normal subjects, and successively 80, 70, 60, 50, and 40 for the five pathological defects). The different levels of heterogeneity and focal defects were validated by physicians so that the simulations were as realistic as possible compared to clinical practice. A total of 42 phantoms were simulated: (1 normal + 6 heterogeneity levels) × (1 normal + 5 TP cortical defect levels). Heterogeneity values (0 to 10,000) and perfusion defect values (93 to 40) were considered by physicians to cover the range of CBF observed in clinical practice.

#### Simulation of the acquisition and reconstruction process

**Figure 2:**
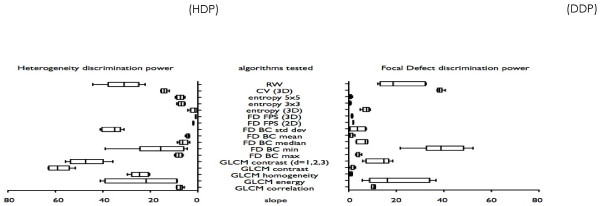
Diffuse heterogeneity and focal defect discrimination power are plotted for each algorithm tested (box and whiskers (mean, median, and min-max values)).

The 42 phantoms were successively projected in 64 projections of 128 × 128. The projections were smoothed with a constant Gaussian kernel rather than a distance dependent function to simulate the point spread function of our gamma-camera (DST-Xl, GEMS, Buc, France) equipped with low-energy ultra-high resolution collimators (LE-UHR, GEMS, Buc, France), corresponding to a full width at half maximum of 5.5 mm for the tomographic acquisition. Poisson noise was added to the projections to simulate the radioactivity emission of the tracer. For each phantom, the total number of counts was equal to 10 M. SPECT images were reconstructed using filtered back projection with a Hamming filter (α = 0.5), which led to 42 reconstructed SPECT exams corresponding to a set of seven levels of diffuse heterogeneity and six levels of focal defects. For the statistical analysis, the diffuse heterogeneity level and the focal defect level were normalized to zero, a normal level, and one, which was the most abnormal level (i.e., 40 for the ‘highest’ focal defect level and 10,000 for the highest heterogeneity level). This scaling is integrated in Figure 2. The Poisson noise was repeated five times, mimicking five different acquisitions of each of the 42 simulated phantoms, which resulted in a total of 210 different simulations. All the 210 simulations were used in the statistical analysis and computed with all methods.

To study only cortical activity, all phantoms were masked. The outward and inward boundaries of the cortical rim were determined by using a threshold of 80% and 55%, as previously described in Volkow et al. [11]. The right side of the mask was generated symmetrically from the left side.

After reconstruction, the heterogeneity quantification parameters, as described above, were computed for each of the 210 phantoms. The results of each algorithm were normalized for a statistical comparison of the discrimination power (a maximal result was a value of 100).

#### Comparison statistics design: discrimination powers

**Figure 3:**
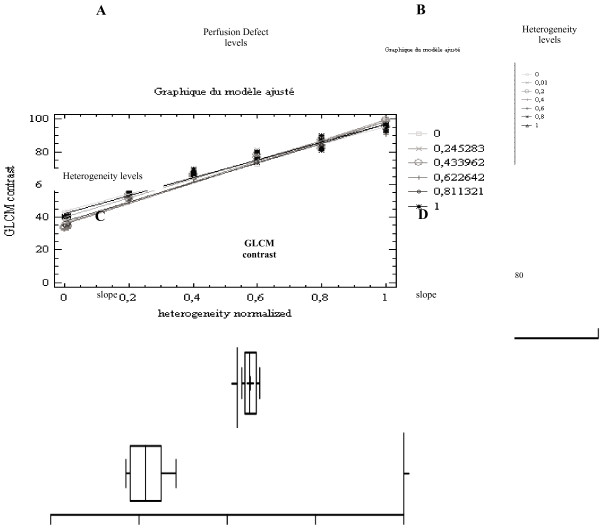
**Diffuse heterogeneity discrimination power and perfusion defect levels.** (**A**) For each heterogeneity level, the GLCM contrast parameter results are plotted as a function of the perfusion defect levels, leading to seven regression lines (0.7 < R^2^ <0.9). (**B**) For each perfusion defect, the GLCM contrast parameter results are plotted as a function of the heterogeneity levels, leading to six regression lines (0.7 < R^2^ < 0.9). (**C**) Box and whiskers (min - max) representation is shown. The mean slope (absolute value) is 1.22, which corresponds to a poor perfusion defect discrimination power (DDP). (**D**) The mean slope (absolute value) is 58.48, which corresponds to a relatively good HDP.

For each algorithm, a diffuse heterogeneity discrimination power (HDP) was obtained as follows: for each level of the six TP perfusion defect, a regression line was calculated between the heterogeneity level and the parameter of the algorithm studied (Figure 3A). The slope of each regression line was calculated. The mean and standard deviation of the slopes were plotted for comparison. The mean slope of the six slopes obtained from a linear regression was considered to be the discrimination power of the algorithm studied for the diffuse heterogeneity quantification (Figure 3C). A mean slope of 1 denotes a maximal separation between the levels of diffuse heterogeneity. A smaller standard deviation (i.e., identical slopes for one method) shows that the heterogeneity quantification is more independent of the TP perfusion defect levels.

A focal defect discrimination power (DDP) is obtained in a same way than the diffuse heterogeneity discrimination power for every algorithm. For each of the seven heterogeneity levels, a regression line was calculated between the perfusion defect level and the parameter of the algorithm studied (Figure 3B). The slope of each regression line was calculated. The mean and standard deviation of the slopes were plotted to compare the methods. The mean of the seven slopes obtained by a linear regression was considered to be the discrimination power of the algorithm studied for the TP perfusion defect level (Figure 3D). A mean slope of 1 denotes a maximal separation between the levels of TP. A smaller standard deviation (i.e., identical slopes for one method) shows that the heterogeneity quantification is more independent of the TP perfusion defect levels.

Mean slopes and range of slopes (i.e., standard deviation) are used as box and whiskers to compare all algorithms (Figure 2). The best heterogeneity quantification algorithm is the one with high HDP (mean slope) and low DDP. Moreover, we are searching the algorithm with HDP as less as possible influenced/correlated with the defects levels (i.e., low standard deviation).

### Subject study

#### SPECT population, acquisition, reconstruction, and display

The heterogeneity quantification parameters were applied to 25 ECD SPECT images from subjects with suspected focal defects, including Alzheimer’s disease, frontotemporal dementia, and primary progressive aphasia (8 women and 17 men; mean age ± SD, 63.6 ± 7 years), and from 15 normal subjects (6 dementia women and 9 men; mean age ± SD, 51.1 ± 13.7 years) acquired in a normal prospective study (CIC-INSERM 02-112-HP). All normal subjects had given their written informed consent to participate in the study. The ethical board of the Medical Faculty of Rouen approved the SPECT procedure. For these normal subjects, the clinical, neurological, and neuropsychological examinations (i.e., the Mini Mental Score Examination, the Mattis Dementia Rating Scale, the Grober-Buschke verbal memory test, the Montgomery-Asberg Depression Rating Scale and Goldberg’s depression test) remained normal. The MRI imaging was normal in these 15 control subjects.

SPECT acquisitions were performed in all 40 subjects with a dual-headed gamma camera (DST-XL, GEMS, Buc, France) equipped with LE-UHR collimators. Acquisitions were performed 1 h after an intravenous administration of 1,000 MBq of 99mTc-ECD under low light and sound conditions. The subject's head was safely positioned in an adjustable head holder. For each SPECT acquisition, 64 angular views of 60 s each were obtained through a 360° circular orbit (32 angular views per head). The data were recorded in a 128 × 128 matrix. The SPECT images were reconstructed from the projection data using the filtered back-projection algorithm with a Hann filter (α = 0.5) and a software zoom of 2 (matrix 128 × 128 × 128, voxel size = 1.7 × 1.7 × 1.7 mm). No attenuation correction was performed. The acquisition and reconstruction parameters used are best-adapted for standard brain SPECT studies in clinical practice [49]. The French Sopha Rainbow look-up table (LUT) (Vision Workstation GEMS, Buc, France) was used for the display of brain SPECT exams. The display was done without high or low thresholding. The cerebellum was considered as the region of reference for the LUT normalization. Brain perfusion exams were presented to physicians in 16 slices (4 × 4) set from the top of the cortex to the bottom of cerebellum. Reconstructed images were normalized in the Talairach space using SPM2 [50].

#### Physicians’ SPECT image interpretation and random walk calculation

The 40 reconstructed images were blindly analyzed and ranked by two physicians according to the degree of diffuse heterogeneity but not according to their focal defect level. The correlation of the ranking of the two observers was calculated. The final ranking was obtained after consensus of the two observers. For each of the five algorithm families, a diffuse heterogeneity parameter was calculated for all SPECT data for 40 slices ranging from AC-PC-10 to AC-PC + 30 after applying both a spatial normalization and a cortical mask.

#### Statistics

The rank of each subject, as defined by the physicians’ consensus, was correlated with the rank provided by each of the five algorithm families with a Spearman coefficient (*ρ*). A statistical significance level of 0.0001 was chosen after Bonferroni correction.

## Results and discussion

### Results

#### Phantoms study

The results of the HDP and DDP for each algorithm are plotted in Figure 2.

The three algorithms that discriminated higher heterogeneity levels (i.e., a high HDP) and were least influenced by focal defects (i.e., a low DDP) were the following: the contrast of GLCM parameter had an average HDP of 58.5 (range from 51.6 to 63) and a DDP of 1.2 (0.2 to 2.9); the standard deviation parameter of fractal dimension box counting (FDBC) had an HDP of 35.9 (31.1 to 41.2) and a DDP of 3.8 (0.4 to 7.4); and the RW algorithm had an HDP of 31.6 (22.2 to 44.3) and a DDP of 20.6 (12.3 to 32.6).

#### Patient study

The correlation between the two physicians’ ranking was *ρ* = 0.86. The correlation of ranking between every algorithm and the physicians’ consensus is presented in Table 1. The RW algorithm shows a significant correlation with the physicians’ consensus (*ρ* = 0.85). The standard deviation parameter of the FDBC, like the GLCM contrast parameter ranking, was not significantly correlated with the physicians’ consensus (*ρ* = −0.46 and *ρ* = −0.1, respectively).

**Table 1:** The correlation (Spearmann coefficient) between the algorithms and the physicians’ consensus

		**Consensus**	**CV**	**Entropy**		**FD**			**GLCM**					**RW**
		**Consensus**	**CV (3D)**	**(3D)**	**3x3**	**BC standard deviation**	**FPS (2D) mean**	**FPS (3D)**	**Contrast**	**Homogeneity**	**Energy**	**Correlation**	**Contrast 3 N**	**RW**
Consensus	Consensus	1	−0,549	−0,12	0,099	−0,458	−0,321	−0,259	−0,099	0,442	0,423	0,16	0,07	0,826 *
CV	CV (3D)		1	−0,013	−0,368	−0,023	0,446	0,194	−0,046	0,73 *	−0,459	0,033	−0,323	−0,558
Entropy	3D			1	0,864 *	0,024	−0,082	−0,404	−0,435	0,195	0,207	0,351	−0,364	−0,14
	3x3				1	0,122	−0,199	−0,177	−0,108	−0,259	0,183	0,032	0,029	0,075
FD	BC standard deviation					1	−0,024	0,295	0,436	−0,227	−0,246	−0,431	0,38	−0,658 *
	FPS (2D) mean						1	0,227	0,274	−0,294	−0,369	−0,243	−0,093	−0,211
	FPS (3D)							1	0,8*	−0,258	−0,725 *	−0,8 *	0,627	−0,197
GLCM	Contrast								1	−0,523	−0,599	−0,936*	0,872*	−0,145
	Homogeneity									1	−0,05	0,522	−0,731 *	−0,312
	Energy										1	0,692 *	−0,421	0,339
	Correlation											1	−0,91	0,167
	Contrast 3 N												1	0,012
RW	RW													1

Correlations with an asterisk correspond to a significant *p* value < 0.0001 after a Bonferroni correction.

The ranking of the 3D and local entropy is significantly correlated (*ρ* = 0.86). Some parameters of GLCM are correlated to each other (contrast and contrast 3 N, for example; *ρ* = 0.87).

## Discussion

Several algorithms from the literature were compared with the original RW algorithm for heterogeneity quantification purposes. These algorithms represented a range of approaches for the possible quantification of diffuse brain SPECT perfusion heterogeneity.

The first comparison was achieved on 210 simulated brain SPECT perfusion exams based on the Zubal head phantom. The major simulation characteristic was the seven different heterogeneity levels mixed with six different regional defects levels. There is no gold standard in defining a brain perfusion heterogeneity pattern. Because this definition is lacking, perfusion heterogeneity was simulated with a Gaussian noise of zero mean distributed on the gray matter. The extreme level of heterogeneity (σ = 10,000) was defined by the physicians. The five levels of heterogeneity were gradually obtained between the normal and extreme pathological level. The GLCM contrast parameter was the more robust algorithm for quantifying the diffuse heterogeneity levels without an influence from the focal defects. The RW algorithm provided relatively good results, but it was influenced by two simulation characteristics, heterogeneity, and focal defects. The influence of the size and intensity of the defect and acquisition quality (count level) have been previously tested [28]. Briefly, the minimal count level acquired must be five million for constant RW measurements. The increase of the size and intensity of the defect result in the increase of the RW results. The increase of heterogeneity (simulated noise) induces an increased in the RW results. Most methods (other than RW and GLCM) are sensitive to focal defects since these are based on global rather than local measurements.

The second comparison was achieved by ranking a set of 40 brain SPECT perfusion exams by their diffuse heterogeneity. We have compared the ranking from the different algorithms and the ranking achieved from the physicians’ consensus. We have shown that the RW algorithm is the only one correlated to the physicians’ consensus. In this case, the GLCM contrast parameter was not correlated with the physicians’ consensus.

For the phantom study, we opted for an analytic simulation method. Our modeling takes into account both Poisson noise in projections and loss of spatial resolution (constant Gaussian rather than a distance dependent function). This kind of analytic simulation method was previously described in Aubert-Broche et al. [51]. Other authors have used Monte Carlo simulations to create brain perfusion SPECT images [52]. Monte Carlo simulations are more realistic because they model stochastic aspects related to photon emission, propagation, and interaction. Nevertheless, we believe that this improvement in the realism of the simulation process should not change the performance of the method tested.

No attenuation correction has been done on patients and phantom studies. The brain SPECT images are not corrected from attenuation in our clinical practice. Therefore, we decided not to test this parameter in this work. The attenuation correction of the brain SPECT images could modify the results of all methods. Some of them may have different sensitivity regarding the attenuation correction.

The results of the two comparison studies seem contradictory. The GLCM contrast parameter provided good results for the simulated images but was not correlated to the physicians’ ranking of the real brain SPECT perfusion images. In fact, the contradiction could be ignored because two different characteristics were tested. In the algorithm comparison for the phantom images, we looked at which algorithm discriminated the most different levels of heterogeneity. We also looked at whether this algorithm is influenced by the perfusion defects. Furthermore, these simulated pathologies were perfectly quantified. In the comparison of the algorithms for the patient population, we looked for an algorithm that would rank the population in the same order as physicians for a population likely to present with focal defects but little diffuse heterogeneity.

The RW method and the physicians’ ranking were significantly correlated (*ρ* = 0.85). It was asked to the physicians to rank the simulated phantoms only regarding the heterogeneity, excluding the intensity of the perfusion defect. The RW method was influenced by the perfusion defects in the diffuse heterogeneity classification in an almost equivalent manner to the physicians’ ranking. However, we demonstrated that RW was influenced by heterogeneity, but also with perfusion defect (Figure 2). A detailed design, behavior, and properties (influence of the two pathologies) of the RW algorithm are provided in an earlier published article [27]. The two physicians were experts in neuro-scintigraphy with almost 10 years of experience each and did not know the different levels of pathology on the phantom set. The physicians classified the patient as normal or abnormal and not (sometimes) clearly as heterogeneous or perfusion defects. When physicians ranked simulated phantoms only by heterogeneity level, they were probably influenced by the abnormality of images caused by perfusion defect. Characterizing the two pathologies at the same time and distinguishing the level was probably a difficult task. The correlation coefficient between the two physicians’ ranking (*ρ* = 0.86) showed that the consensus was necessary for a final decision, and a tool to reduce inter- and intra-observer bias for diffuse heterogeneity quantification is needed. This point demonstrates a limitation in the RW method. This limitation of the RW algorithm looks similar to the limitation found in clinical interpretation, but is an advantage if the aim is to obtain a similar ranking with the physicians.

The GLCM contrast parameter was not influenced by the focal defects and discriminated diffuse heterogeneity well. Therefore, it was expected that the classification of the population by this algorithm was far from the medical classification.

## Conclusions

In conclusion, the patient study demonstrated the following: (1) The definition of a normal SPECT exam is complex because normal subjects can be classified as clinically heterogeneous apart from all other clinical data. (2) It is a difficult task to rank patients with extended focal hypoperfusion as patients not having a diffuse heterogeneity.

The phantom and patient study results demonstrated the following: (1) The RW method was correlated with the medical classification. However, an algorithm, such as GLCM contrast parameter, provided better results than the RW method for the diffuse heterogeneity classification when heterogeneity was strictly defined as a phenomenon that resembles noise (coming from the acquisition or reconstruction process). (2) Both RW and physician’s results appear to reflect both heterogeneity and presence of lesions rather than heterogeneity alone.

These results must be confirmed by future studies of patients with diseases that have only diffuse heterogeneity and no focal defects.

## Competing interests

The authors declare that they have no competing interests.

## Authors’ contributions

TL and EJ participated in the design of the RW algorithm. RM drafted the manuscript, designed the phantom and patient study and performed the statistical analysis. PV conceived of the study, and participated in its design and coordination. All authors read and approved the final manuscript.
